# Autocatalytic Loop, Amplification and Diffusion: A Mathematical and Computational Model of Cell Polarization in Neural Chemotaxis

**DOI:** 10.1371/journal.pcbi.1000479

**Published:** 2009-08-28

**Authors:** Paola Causin, Giuseppe Facchetti

**Affiliations:** Department of Mathematics “F. Enriques”, Università degli Studi di Milano, Milano, Italy; California Institute of Technology, United States of America

## Abstract

The chemotactic response of cells to graded fields of chemical cues is a complex process that requires the coordination of several intracellular activities. Fundamental steps to obtain a front vs. back differentiation in the cell are the localized distribution of internal molecules and the amplification of the external signal. The goal of this work is to develop a mathematical and computational model for the quantitative study of such phenomena in the context of axon chemotactic pathfinding in neural development. In order to perform turning decisions, axons develop front-back polarization in their distal structure, the growth cone. Starting from the recent experimental findings of the biased redistribution of receptors on the growth cone membrane, driven by the interaction with the cytoskeleton, we propose a model to investigate the significance of this process. Our main contribution is to quantitatively demonstrate that the autocatalytic loop involving receptors, cytoplasmic species and cytoskeleton is adequate to give rise to the chemotactic behavior of neural cells. We assess the fact that spatial bias in receptors is a precursory key event for chemotactic response, establishing the necessity of a tight link between upstream gradient sensing and downstream cytoskeleton dynamics. We analyze further crosslinked effects and, among others, the contribution to polarization of internal enzymatic reactions, which entail the production of molecules with a one-to-more factor. The model shows that the enzymatic efficiency of such reactions must overcome a threshold in order to give rise to a sufficient amplification, another fundamental precursory step for obtaining polarization. Eventually, we address the characteristic behavior of the attraction/repulsion of axons subjected to the same cue, providing a quantitative indicator of the parameters which more critically determine this nontrivial chemotactic response.

## Author Summary

The ability of cells to respond to chemical signals present in the environment is of upmost importance for life. In the developing embryo, cells crawl along graded fields of chemical cues to aggregate into organized patterns. This process is an example of chemotaxis. It is a complex phenomenon, where external signals are transduced into internal chemical pathways leading to directional movement. Differential reorganization of the internal structure is called polarization, and it involves regulatory proteins as well as cytoskeletal elements. In this work, we propose a mathematical and computational model for the quantitative study of chemotactic pathfinding in neural cells. Our starting point is the recent finding that, for such cells, an early polarization event is the redistribution on the membrane of cue–ligated receptors, transported by the cytoskeletal structures, which act as a sort of conveyor belt. We show that this proposed mechanism, connecting in a closed loop cue sensing and cytoskeleton dynamics, is qualitatively and quantitatively adequate to produce polarization. We also investigate the role of the internal biochemical chain in producing signal amplification and its tight interlacing with polarization. An extension of the model is used to study chemotactic behaviors as the attractive/repulsive response of axons exposed to the same cue.

## Introduction

Eukaryotic cells like neutrophils or amoebas migrate in response to chemical stimuli. External graded signals are transduced into internal pathways, giving rise to cell front–back asymmetrization, which leads to cytoskeleton reorganization and, eventually, directional motion. The process by which regulatory proteins and other molecular species, initially uniformly distributed in the membrane and cytosol, differentially localize is called polarization and is a steering event in the chemotactic response. Polarization in eukaryotic cells has been the object of intense *in vivo* and *in vitro* studies (see, *e.g.*, [1]–[3] and references therein). *In silico* models represent a third viable approach to explore this complex phenomenon. A mechanism of polarization advocated in several computational models is the interplay in the cell of a local activator–global inhibitor effect (the so–called LEGI mechanism, see, *e.g.*, [4]–[6]); alternative mechanisms have also been envisaged, which do not require the introduction of the global inhibitor, which is a somewhat controversial topic, but generate front–back asymmetrization relying on some sort of interaction – say, competition, coincidence of action, feedback – between different pathways (see, *e.g.*, [1], [7]–[10]).

In this paper, we propose a mathematical and computational model which addresses polarization in the far less studied case of neural cells. We focus on axon chemotactic pathfinding, a fundamental process for the innervation of synaptic targets in the developing embryo. In order to perform turning decisions, axons develop front–back polarization in their distal structure, the growth cone (GC). Internal polarization differentially induces microtubule protrusion or collapse in the GC cytoskeleton determining directional migration [11]. Mathematical models in this context most often do not enter into the details of the biochemical signalling cascade, but rather adopt phenomenological simplified descriptions that provide a “black box” information of the functional behavior of the system. A first class of approaches (see, *e.g.*, [12],[13]) is based on persistent random walk models. The GC trajectory is typically described by a system of ordinary differential equations accounting for a deterministic velocity field and random “kicks” arising from stochastic terms, macroscopically representing fluctuations in gradient sensing and signal transduction. Evolutions of these models are presented in [14] and further in [15], where the GC trajectory is described by more sophisticated stochastic partial differential systems of equations, including diffusion and inertia contributions. A second class of models are investigated in [16]–[18], where there is the attempt of introducing a description of the intracellular chain. Namely, the probability of finding a transmembrane receptor at a certain angular position on the GC is supposed to be linked to some significant intracellular parameter, for example the local concentration of ionic calcium [16]. This latter approach is prodromal to the present study of the spatial organization of the GC during guidance.

The biophysical starting point of the present work is the experimental finding, recently obtained by Bouzigues et al. [19], that GC receptors subjected to the graded field of an attractant cue undergo two fundamental types of motion on the membrane: free diffusion, which is always present -also under an uniform external field- and drift motion. This latter kind of motion interestingly makes neural cells different from nonneural ones, whose receptors seem to undergo both in uniform and graded fields an unbiased continual diffusion [20]. Due to the drift motion, GC receptors rearrange on the membrane asymmetrically concentrating on the side facing the source. The redistribution is driven by the physical interaction of bound receptors with microtubules, which serve as conveyor belts [21]. An autocatalytic loop is then established: bias in receptor localization induces, via internal polarization of molecules, preferential growth of the microtubules toward the leading edge of the GC. This, in turn, enhances convey of receptors on that same side. In order to mathematically investigate the sustainability of this hypothesis of receptor autocatalytic loop, we model the chemical pathway triggered upon receptor binding. In such a pathway, cyclic nucleotides act as second messengers. Their role is monitored with particular attention in the simulation, since they are known to be key–regulators of GC motility [22]. In particular, whole cell recordings of inward calcium currents at *Xenopus* spinal neuron GCs indicate that cyclic nucleotide signalling modulates the opening of L–type voltage–dependent calcium channels (LCC) [23],[24]. We make here the modelling assumption that the driving force which induces biased recruitment of receptors is the uneven distribution (that is, the gradient) of such calcium channels. This is a lumped representation of the complex mechanical events connected with cytoskeleton reorganization induced by calcium dynamics, *i.e.*, actin dynamics, microtubule polymerization and depolymerization [25]. Intracellular reactions constitute the bridge between the upstream receptor and the downstream calcium dynamics. Due to their enzymatic nature, which triggers a one–to–more activation of molecules, they represent an amplification process, fundamentally sustaining polarization. We use the model to obtain quantitative information on this delicate interlacing.

To work on a practical groundfield, we consider here the pathway activated by exposure of GCs to a graded signal of the diffusible molecule netrin, which role as chemoattractant in conjunction with its receptor *Deleted in Colorectal Cancer* (DCC) [26] is well recognized and studied in axon guidance [27]. The model of the intracellular cascade takes as a biophysical reference the pathway proposed in the work by Nishlyama et al. [28], which addresses in a detailed manner netrin–induced signalling. We also refer to the work by Clément et al. [29]: albeit treating a different topic (the regulation of ovarian follicles by the FSH hormone), this work provides a mathematical description of biochemical processes from receptor binding to second messenger cyclic AMP activation, which can be conveniently taken as a starting point also for our case.

Eventually, we propose an extension of the model to perform a quantitative study of a characteristic chemotactic behavior exhibited by GCs. While in nonneural cells transmembrane receptors are a limited and well identified set, receptors for neural navigation are much more heterogeneous. In particular, the emerging picture is that in most cases there are several receptors for each guidance cue and these receptors might work in complexes [30]. Engagement of receptor complexes is involved in the specificity of the response to a cue. The UNC5 receptor [31] forms a complex with DCC via interaction of the cytoplasmic domains. Binding of the complex with netrin converts the attractive into repulsive response [22],[24]. Repulsion arises due to activation by the DCC–UNC5 complex of an alternative pathway leading to the production of cyclic GMP, linked to cytoskeleton collapse [32]. Genetic manipulations can modulate the GC response in presence of different percentages of UNC5 expression, ideally allowing to observe a passage from attraction (for low percentages of the DCC–UNC5 complex) to repulsion (for high percentages of the DCC–UNC5 complex). In [28], it is proposed that a synthetic parameter that describes in this framework the chemotactic response may be represented by the ratio between the average concentrations of cyclic AMP and GMP: high ratios would favor attraction, whereas low ratios favor repulsion. We use our model to quantitatively assess the significance of such an hypothesis, highlighting a possible mechanism of synergistic interaction of the two pathways, which provides an interpretation of the chemotactic response.

### Mathematical Model

Growth cones are 3D hand-shaped structures, which dynamically change their conformation. Filopodia protrude from the GC membrane, continuously extending and retracting to explore the environment and to create adhesion to the substratum. These phenomena are highly complex and take place at a time frequency faster than the one we are interested into, typically seconds vs. a few minutes. For the purposes of the present model, we neglect fine local details of the shape and we represent GCs as 2D disk-like structures with diameter of 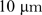. This simplification models the average shape assumed by GCs in their state prior to actual motion (the “pausing state” cited in [19]). The same assumption has been made by other authors interested in GC mathematical modelling, see [16],[33],[34]. We refer as a paradigm to the *in vitro* chemotactic assay for neural cells, where GCs are exposed to steady graded concentrations of a chemical ligand released by a pipette (see, *e.g.*, [35],[36]). Observe that this is essentially a two–dimensional situation.

**Figure 1:**
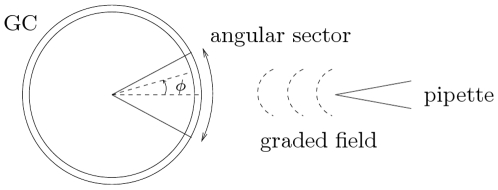
GC schematization. The GC is represented in the mathematical model as a disk subdivided in angular sectors. We consider the settings of the chemotactic assay for neural cells, where a pipette (here on the right) establishes a steady graded field of a chemotropic molecule.

Polar coordinates are used, the origin of the axes being positioned in the center of the GC. The angle 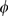 denotes the azimuthal coordinate on the membrane and its origin is set along the direction connecting the GC center with the pointwise source, which we always suppose to lay on the right hand side of the cell (see Fig. 1). We denote by 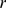 the radius of the GC.

In the model, we deal with membrane species (receptors, cyclases and calcium channels) and cytosolic species (G–proteins, cyclic AMP, cyclic GMP, kinases and their corresponding precursors). To connect membrane and cytosolic concentrations (and vice versa), we use the dimensional corrective factor 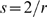 (or its inverse). We use the notation 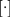 to represent concentration of a certain species; moreover, we denote by the superscript 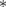 the bound or active form of the molecules. The nomenclature for the species is reported in Tab. 1.

**Table 1:** Nomenclature of the species entering the mathematical model.

Symbol	Definition
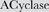	inactive adenylate cyclase
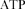	adenosine triphosphate
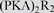	pre–kinase protein in cAMP pathway
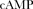	cyclic adenosine monophosphate
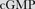	cyclic guanosine monophosphate
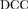	DCC receptor
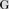	G–protein in the DCC pathway
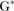	activated G–protein
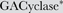	G–activated adenylate cyclase complex
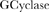	inactive guanylate cyclase
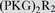	pre–kinase protein in cGMP pathway
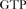	guanosine triphosphate
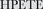	12-hydroxyperoxyeicosatetraenoic acid
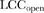	open 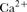 L-channel
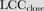	close 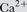 L-channel
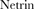	extracellular signal netrin
PDE	phosphodiesterase
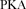	adenosine–dependent kinase protein
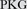	guanosine–dependent kinase protein
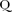	G–protein in the UNC pathway
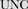	DCC–UNC5 receptor complex

#### DCC-activated biochemical pathway

**Figure 2:**
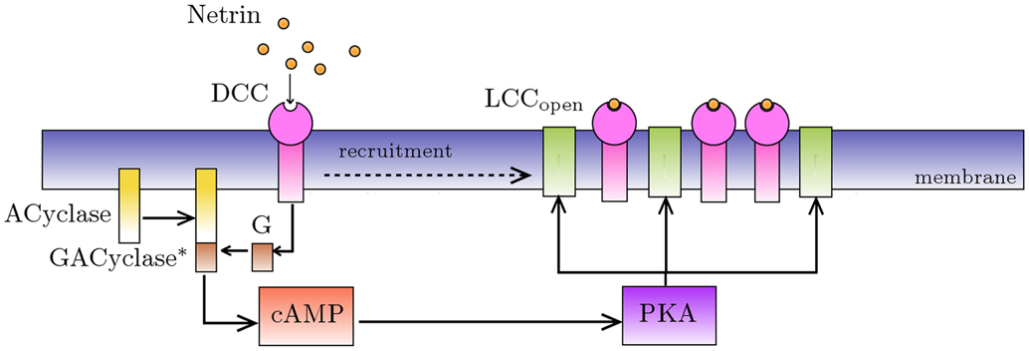
Proposed path for GC chemotaxis induced by netrin binding with DCC receptors. Solid arrows indicate the prevalent direction of chemical reactions, the dashed arrow indicates physical displacement (recruitment) of receptors induced by open calcium channels.

In the following, we connect receptor ligation to calcium channel dynamics by proposing a biochemical pathway, where elevation of intracellular cAMP level is a pivotal event. We refer to Fig. 2 for a schematic representation of such a pathway.

Binding of netrin to its receptor DCC results in the formation of the ligated complex 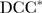


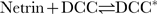
The activated site of the bound DCC receptor on the intracellular side catalyzes the release of the guanosin-diphosphate (GDP) contained in the cell G protein (for example Rac1, CDC42 or RhoA) and its substitution with a molecule of guanosin-triphosphate (GTP) [37]

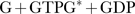
The released 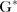 combines with the membrane protein adenylate cyclase (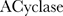)
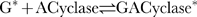
which, in turn, enzymatically catalyzes the synthesis of cAMP from the available substrate ATP
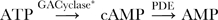
cAMP molecules bind the substrate prekinase protein (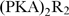, R being the regulatory subunit) to give the active kinase adenosine-dependent protein (PKA)
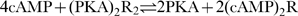
which enzymatically activates the opening of LCC channels
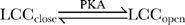



#### Diffusive and advective fluxes

Let 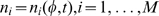 be a generic chemical species of the model. Each chemical species is subjected to Brownian diffusion which causes its spreading in a homogenization process. In the present model, we only consider lateral diffusion of the species, neglecting radial diffusion (see [7],[9] for similar hypotheses and discussions on this subject).

We model the Brownian diffusive flux 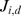 of the species using the standard Fick's law
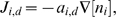
(1)

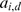
 being the diffusion coefficient.

The directional motion of bound receptors due to the interaction with microtubules mathematically represents a drift term. In the lumped description we adopt, the convective field is the lateral gradient of open calcium channels. The form of the flux 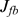 due to feedback we consider reads
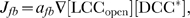
(2)where 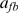 is a multiplicative coefficient, and where we have supposed the feedback flux to be an increasing function of the concentration of bound receptors (see [38] for a similar modelling assumption in a more general context). Note that the minus sign in (1) represents the fact that diffusion smooths away concentration gradients, while the positive sign in (2) indicates motion toward the convective field, here the increasing gradient of calcium channels (positive feedback). This latter effect is shown in Fig. 2, where we have represented with a dashed arrow the displacement of receptors induced by open calcium channels.

The total flux of molecules of the 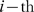 species is then given by
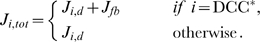



#### Model equations for the DCC signal transduction pathway

Conservation laws give the rate of change of concentrations of each species. For the generic species 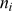, we have
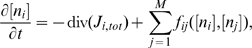
(3)where the nonlinear functions 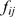 at the right hand side account for reaction terms arising from the application of the mass action law to the chemical reactions in the above described pathway. The following system of partial differential equations of the form (3) represents our model for the DCC signal transduction pathway: given the appropriate initial and boundary conditions, solve for time 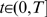


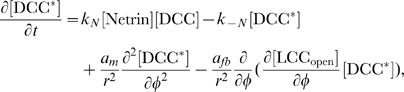
(4a)

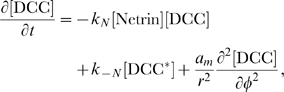
(4b)

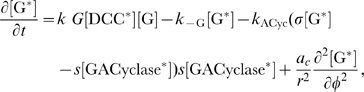
(4c)

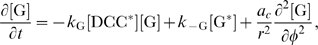
(4d)

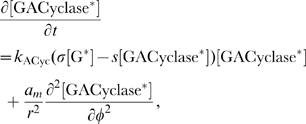
(4e)

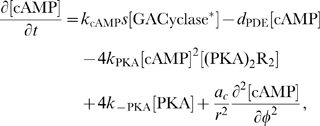
(4f)

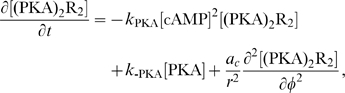
(4g)

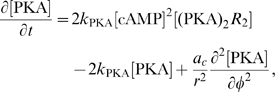
(4h)

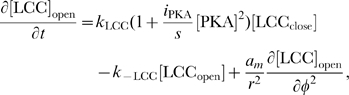
(4i)

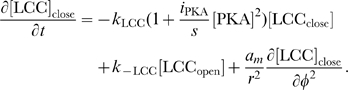
(4j)


Note that in the case of the activation of adenylate cyclase (Eq. (4–c)), where a complex protein conformational change occurs, we have used a “physiological” and not a biochemical representation, introducing a logistic–type law as in [29]. The 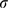 coefficient in this term is an amplification parameter which represents the average number of adenylate cyclase molecules activated by one 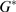 molecule.

We refer to the Methods Section for a discussion on the choice of initial and boundary conditions and of the kinetic parameters.

### Attractive vs. Repulsive Behavior: The DCC–UNC5 Complex Pathway

In this section, we propose an extended version of the model to study the bifunctional response to a guidance cue, a phenomenon known to interest the response to netrins [27]. Engagements of receptor complexes is known to control the specificity and the polarity of the response of the neuron to the guidance cue. Here we will not deal with the details of the dynamics of the formation of the DCC–UNC5 complex, and we will always consider such a dynamics at the equilibrium. From the modelling point of view, this amounts to prescribe *a priori* the percentage of DCC receptors forming a complex with UNC5 (on this issue, see also the discussion in the Methods Section). Setting in the tests various percentages, we analyze a wide spectrum of situations. To describe the DCC–UNC5 complex, we follow here the idea proposed in [28], and namely that such a complex leads to cGMP synthesis, regulated by 12-hydroxyperoxyeicosatetraenoic acid (HPETE) via direct activation of guanylate cyclase [39]. Enhancement of the cGMP level causes to calcium channel closure [40]. This fact is at the origin of the significant decrease of inward calcium flux in UNC5–overexpressing GCs, repelled by netrin.

Due to the substantial similarity with the sole DCC pathway, many mechanisms are modeled here in the same way. The binding reaction for the DCC–UNC5 complex (for clarity indicated just as UNC) reads
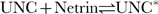
The ligated complex induces dissociation of a G–protein (probably of Rho type, see [12]), that we denote here by 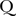, giving
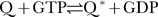
This process leads to the formation of HPETE. Not being yet completely explained, we consider here a second order reaction to occur (see the Methods Section for a more detailed discussion of this aspect)
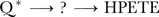
HPETE activates soluble guanylate–cyclase (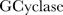)
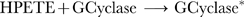
which, in turn, catalyzes the synthesis of cGMP from the guanosine triphosphate (GTP) substrate
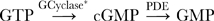
Formation of the guanosine–dependent kinase protein (PKG)
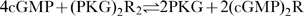
enzymatically enhances closure of calcium channels
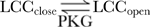



#### Model equations for the DCC-UNC5 signal transduction pathway

The rates of change of concentrations for the DCC–UNC5 pathway are modeled by the following system: given the appropriate initial and boundary conditions, solve for time 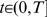


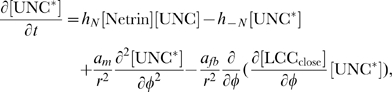
(5a)

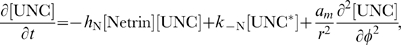
(5b)

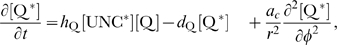
(5c)

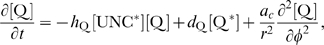
(5d)

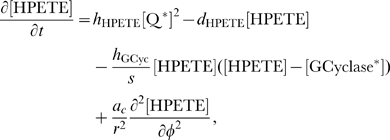
(5e)

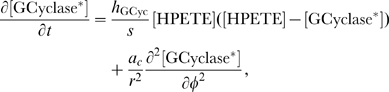
(5f)

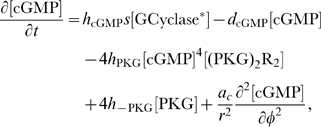
(5g)

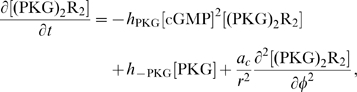
(5h)

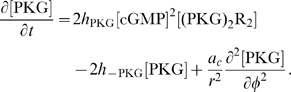
(5i)


The last term at the right hand side of Eq. (5–a) represents the feedback of the calcium dynamics on the redistribution of receptors. Note that here the feedback effect is exerted from closed channels: such a term must not be interpreted from a strict biochemical point of view, but again as a lumped phenomenological description of a more complex mechanism.

**Figure 3:**
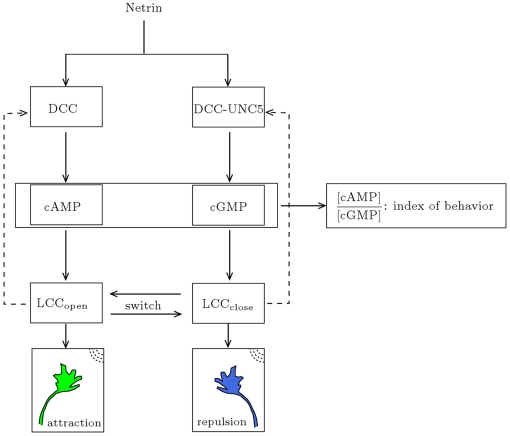
Schematization of the antagonist pathways induced by netrin binding with DCC (left column) and DCC-UNC5 receptor complex (right column). The dashed arrows indicate the feedback effect in the closed loop model.

Equations for the calcium channels still must be added. In the following, we will be interested in the coupling of the DCC and DCC–UNC5 pathways. To model this situation, we consider Eq. (4i) and (4j) modified as
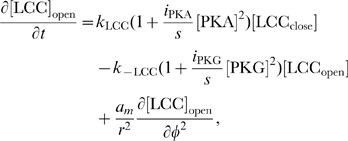
(6a)

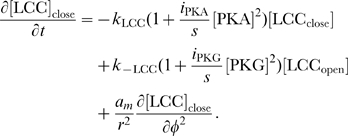
(6b)where now the rate of opening of calcium channels is determined by two competing effects, the enhancing action of the DCC pathway and the inhibitory action of the DCC–UNC5 pathway. Fig. 3 schematically depicts the model of the interaction.

## Results

We refer to Eqs. (4a–j) as the DCC model, while we refer to Eqs. (4a–h),(4a–i) and (4a–b) as the coupled DCC–UNC5 model. In all the tests presented in the following sections, we have assigned a steady exponential profile of netrin concentration with 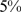 steepness, such that the concentration at the source is equal to 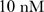 (dissociation constant of netrin [26]).

### DCC Model: Achievement of Polarization

**Figure 4:**
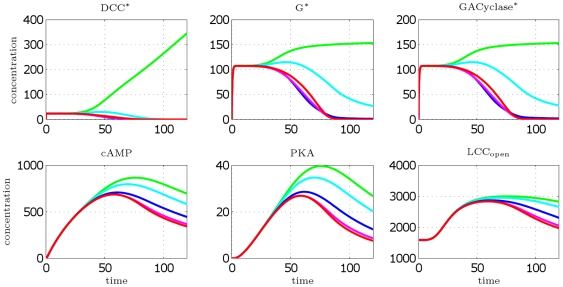
DCC model: concentration profiles as a function of time (in minutes). Curves are relative to the four most representative angular sectors, perceiving a cue concentration ranging from minimal to maximal value, respectively. Legend: green [Netrin] = 9.52 nM (max value), cyan [Netrin] = 9.36 nM, blue [Netrin] = 9.16 nM, magenta [Netrin] = 9.08 nM, red [Netrin] = 9.07 nM (min value).

**Figure 5:**
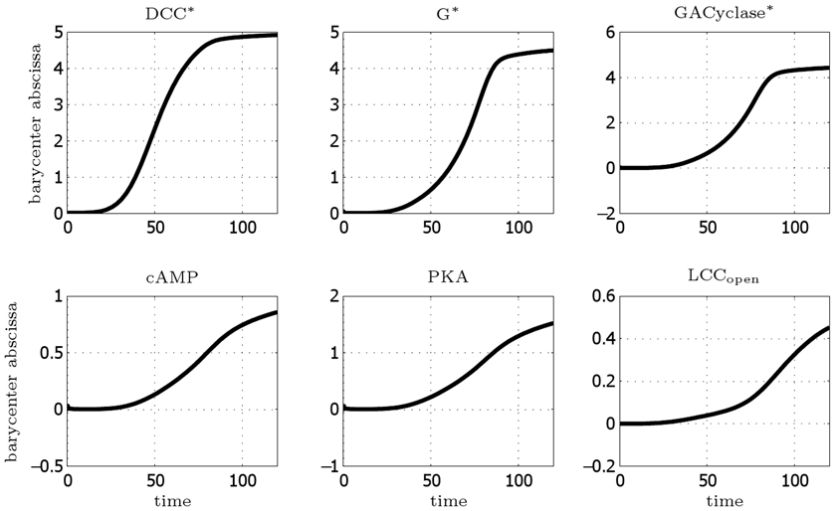
DCC model: abscissa (in [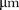]) of the barycenter of the molecules as a function of time (in minutes).

**Figure 6:**
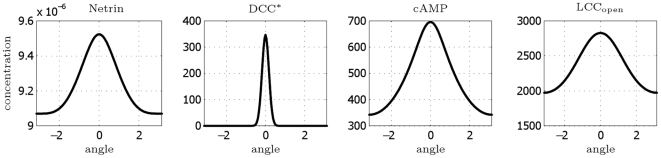
DCC model: concentration profiles of selected species after 2 h as a function of the angle 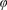.

**Figure 7:**
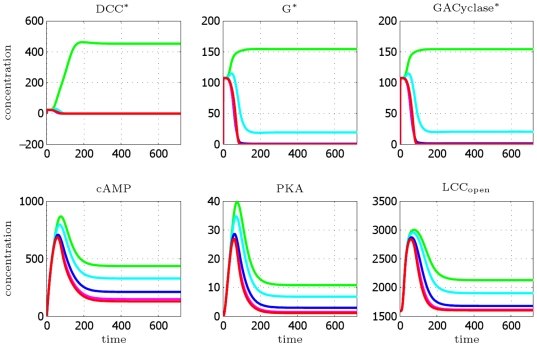
DCC model: concentration profiles as a function of time (in minutes), integration till *T* = 12 h. Curves are relative to the four most representative angular sectors, perceiving a cue concentration ranging from minimal to maximal value, respectively. Legend: green [Netrin] = 9.52 nM (max value), cyan [Netrin] = 9.36 nM, blue [Netrin] = 9.16 nM, magenta [Netrin] = 9.08 nM, red [Netrin] = 9.07 nM (min value).

We first demonstrate that the model correctly achieves polarization and reaches a steady state condition, where front–back differentiation is established. In Fig. 4, we plot the concentration profiles as a function of time, obtained from simulations carried out till 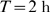. In Fig. 5, we plot for the different species the abscissa of the barycenter of the molecules as a function of time. Since at the initial time the distribution of molecules is homogeneous, the displacement of their spatial barycenter from 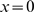 represents an index of the intensity of the polarization. In Fig. 6, we plot the concentrations after 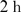 as a function of 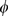. Significant polarization of the receptors takes place in tenth of minutes; polarization is inherited by all the internal species. An interesting behavior is shown by calcium channels, which undergo in all sectors a first phase of opening, reaching a fairly similar maximum value, followed by closure, more pronounced in the rear side. This mechanism might represent a sort of LEGI, global “inhibition” being constituted by collective opening and local “activation” by differential closure. Note that this is to be intended only as a qualitative interpretation (see also the discussion in [11]). The position of the barycenter of bound receptors presents a sigmoid behavior, which is characteristic of autocatalyzing processes: a first phase of relatively slow accumulation (lag time) followed by a quick growth till a steady state. This is in agreement with the experimental result of [19, Fig.1e]. In Fig. 7, we plot the concentration profiles as a function of time for a simulation with a longer integration interval 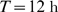. A steady state is definitely reached.

### Contribution of Feedback and Amplification to Polarization

**Figure 8:**
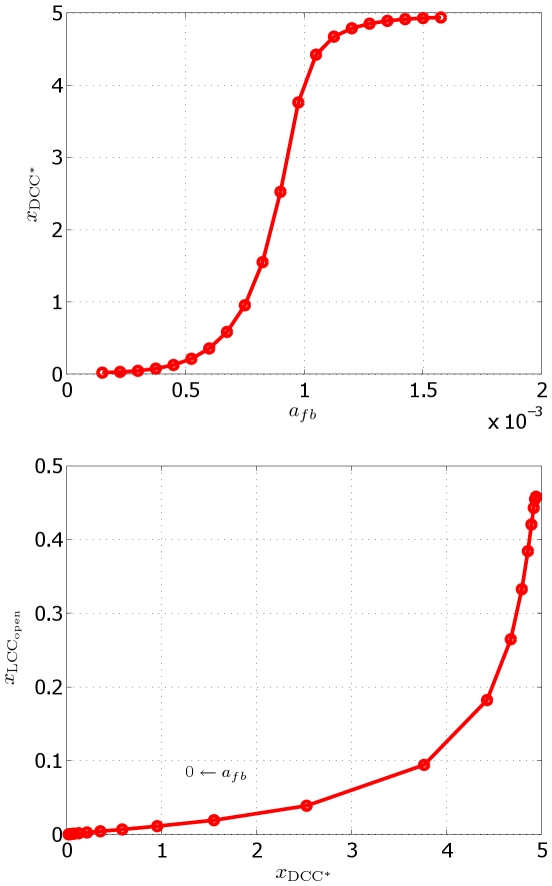
Effect of the variation of the feedback coefficient 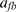. Top: abscissa 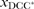 (in 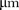) of the barycenter of bound DCC receptors as a function of the feedback coefficient 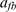. Bottom: abscissa 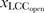 (in 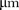) of the barycenter of open calcium channels as a function of the abscissa of the barycenter of bound receptors (in 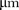). Each marker corresponds to a simulation carried out with a different value of 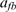.

In [19], an experiment is reported, where a chelator is used to subtract the calcium available in the cytosol, obtaining a suppression in the asymmetric relocalization of receptors. To perform an *in silico* investigation of this experiment, we have studied the effect of the variation of the feedback coefficient 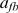. Fig. 8 (left) shows that significant receptor relocalization is obtained only above a nonzero threshold of 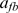. More importantly, the model predicts that the lack of receptor relocalization implies absence of chemotactic response and not only a weaker, but still existing, response. Fig. 8 (right) quantifies how the asymmetry in DCC receptors localization is reflected in downstream differential opening of the calcium channels. This indicates that a sufficient active relocalization of receptors is an upstream enhancing event needed to produce chemotactic response.

**Figure 9:**
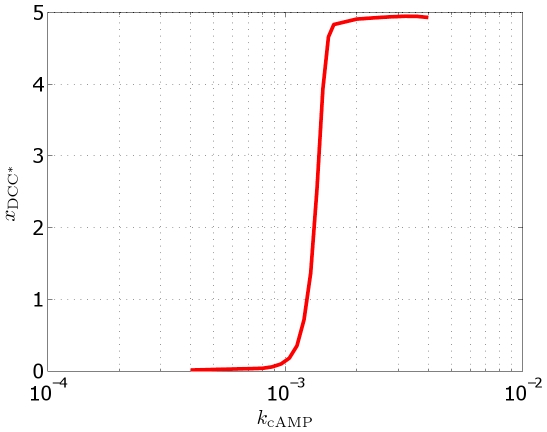
Effect of the variation of the parameter 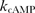. Abscissa (in 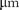) of the barycenter of the bound receptors as a function of 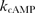, index of the enzymatic amplification. Below a non–zero threshold, no polarization occurs.

In a second investigation, we have considered the effect of amplification due to chemical kinetics. In the signalling pathway, we may identify –loosely speaking– two families of reactions. The first family consists of stoichiometric reactions, as for example ligand–receptor or G–cyclase binding, involving one–to–one molecule synthesis. The second family consists instead of enzymatic reactions, which, involving a one–to–more molecule production, drive an internal amplification processes. We focus our attention on this latter family, taking as a representative case the cAMP production catalyzed by cyclase. We perform different simulations with a decreasing kinetic constant 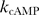, which quantitatively modulates how many molecules of cAMP are produced starting from an available molecule of activated cyclase. In Fig. 9, we plot the position of the barycenter of the bound receptors as a function of 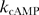. This result suggests that polarization is also indissolubly crosslinked with internal amplification. A parametric constraint appears: below a non–zero threshold in the enzymatic (that is, amplifying) efficiency of the pathway, no polarization occurs.

The above investigations show that both feedback and amplification are precursor events for the achievement of polarization. Their respective actions are necessary and concurring contributions.

### Diffusion vs. Feedback: Antagonist Roles

**Figure 10:**
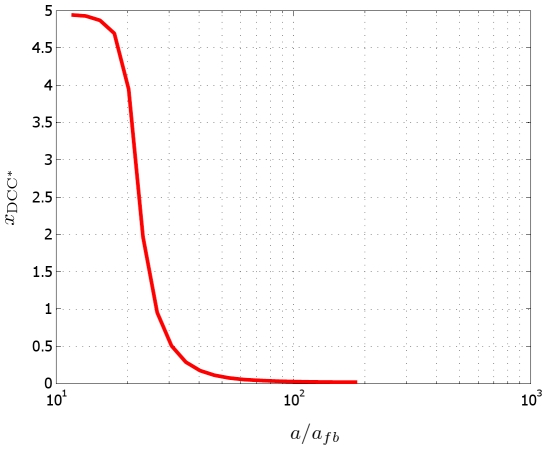
Diffusive vs. convective phenomena. When diffusion overwhelms drift effects, the species are homogenized and polarization is not created. The abscissa of the bounded DCC receptors tends to zero, as if under an uniform chemotropic field.

The results discussed above were obtained setting in the model the diffusion and feedback coefficients as indicated in Tab. 2. While the diffusion coefficient can be measured as a well defined physical parameter, it is much more difficult to quantify the feedback parameter. This fact has a strong implication, since the ratio of the two parameters influences the dominating behavior of the system. To fix the ideas, we perform the tests varying the feedback coefficient, and we keep constant the diffusion coefficients 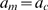, (that we denote here for simplicity as 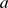). The results of the simulations are shown in Fig. 10, where the abscissa 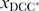 of the barycenter (in 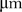) of the bound receptors is plotted as a function of 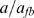. They suggest that under a certain threshold, diffusion overwhelms drift, leading to an unbiased receptor distribution on the membrane, that is 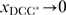, as if the external field were uniform.

**Table 2:** Diffusion and feedback coefficients.

Parameter	Definition	Value	Dimension	Ref.
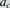	cytosolic diffusion coefficient	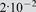	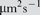	
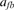	feedback coefficient	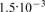	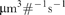	
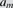	membrane diffusion coefficient	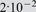	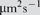	[7]

### Time Scales in Front and Back Biochemical Processes

**Figure 11:**
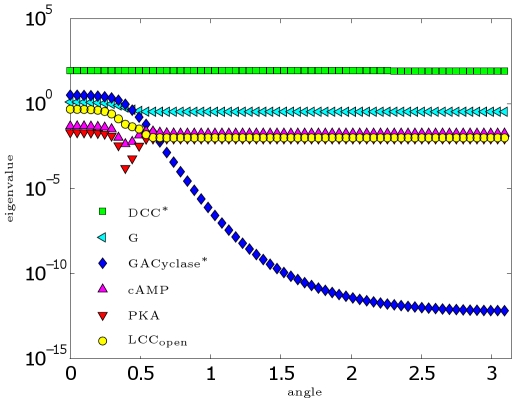
Spectral analysis of the model. Eigenvalues (in absolute value) of the Jacobian matrix of the DCC simplified model at steady state, as a function of the angular coordinate 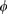 (for symmetry represented here only in the range 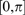).

We use the model to study the time scales that characterize the process on the front and back sides, respectively. To perform this mathematical analysis, we consider a simplified version of the DCC model, neglecting diffusion and feedback terms. By doing so, we yield a system of ordinary differential equations, decoupled sector by sector. In this study, we prescribe *a-priori* an asymmetric receptor distribution to describe the polarized situation reached after a sufficient time of exposure to the cue. In particular, we start from the steady state distribution of receptors obtained from the simulation of the DCC model with 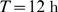. We compute in each sector the eigenvalues of the Jacobian matrix of the system in correspondence to its steady state. All the eigenvalues are real negative, indicating that the steady state is an attractive point. Based on the principal component of the corresponding eigenvector, we associate a chemical species with each eigenvalue. Then, using standard tracking techniques [41], we follow the variation of each eigenvalue along the GC perimeter. In Fig. 11, we plot the modulus of the eigenvalue associated with each species as a function of the angle 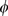. The eigenvalue associated with the slowest process on the front side (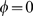) appears to be connected to PKA, while on the back side (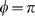) it appears to be connected to 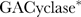. Observe that all the eigenvalues undergo a variation along the angle, even if for some of them this is not apparent in the logarithmic scale, required to appreciate the different relative behaviors. The graph shows the strong variation of the eigenvalue connected with 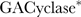. Moreover, the general trend of reduction of the absolute values passing from the front side to the back side indicates that the front dynamics is faster than the back one.

### Coupled DCC–UNC5 Model: Attractive vs. Repulsive Responses

We use the coupled DCC–UNC5 model to study the response of the system to the (bifunctional) netrin cue when the DCC–UNC5 complex is formed. Denoting by #DCC and #UNC the number of receptors on the membrane belonging to the two populations, respectively, we introduce the quantity
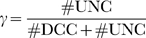
which represents the fraction of DCC–UNC5 receptors. Neurons which display a chemoattractive response when exposed to a netrin gradient are typically characterized by low values of 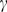. Neurons genetically manipulated to overexpress the UNC5–type receptors, which display a chemorepulsive response when exposed to the same netrin gradient, are typically characterized by values of 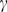 near 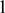.

**Figure 12:**
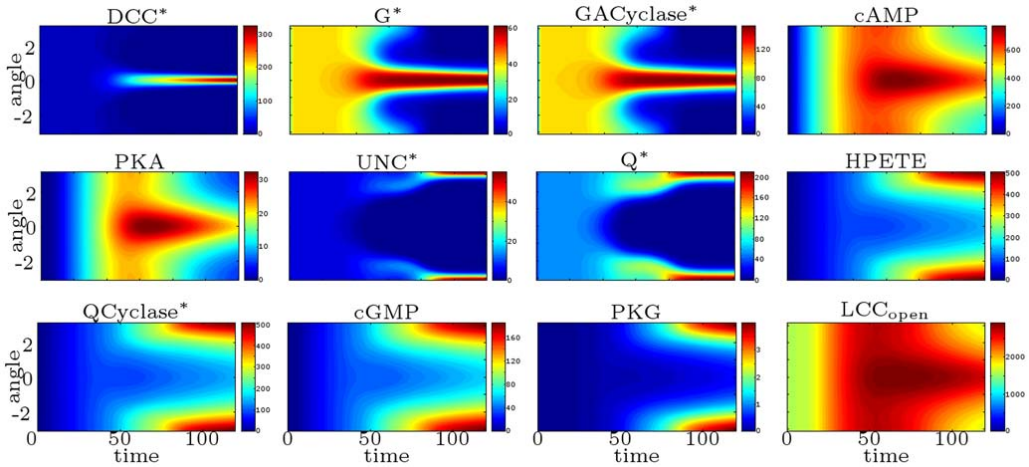
Attractive chemotaxis (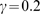). Concentration contours as a function of time (in minutes, 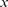 axis) and angular coordinate 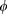 (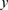 axis).

As a representative situation of the first case, we set 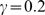. In Fig. 12, we plot the concentration contours of the main species as a function of angular position 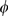 and time. This model suggests the following explanation of the attractive behavior: DCC receptors migrate toward the source, while DCC–UNC5 ones migrate away from it, causing a differential opening of calcium channels on front vs. back.

**Figure 13:**
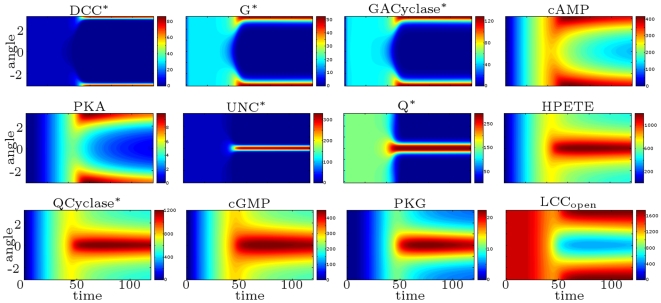
Repulsive chemotaxis (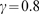). Concentration contours as a function of time (in minutes, 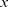 axis) and angular coordinate 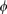 (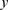 axis).

Then, we consider the dual case, setting 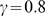. In Fig. 13, we plot the concentration contours of the main species as a function of angular position 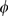 and time. The model suggests a dual behavior with respect to the situation with 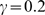: DCC–UNC5 receptors migrate toward the front, while DCC receptors migrate toward the back, giving rise to the repulsive response.

#### Role of second messengers: the cAMP vs. cGMP ratio

**Figure 14:**
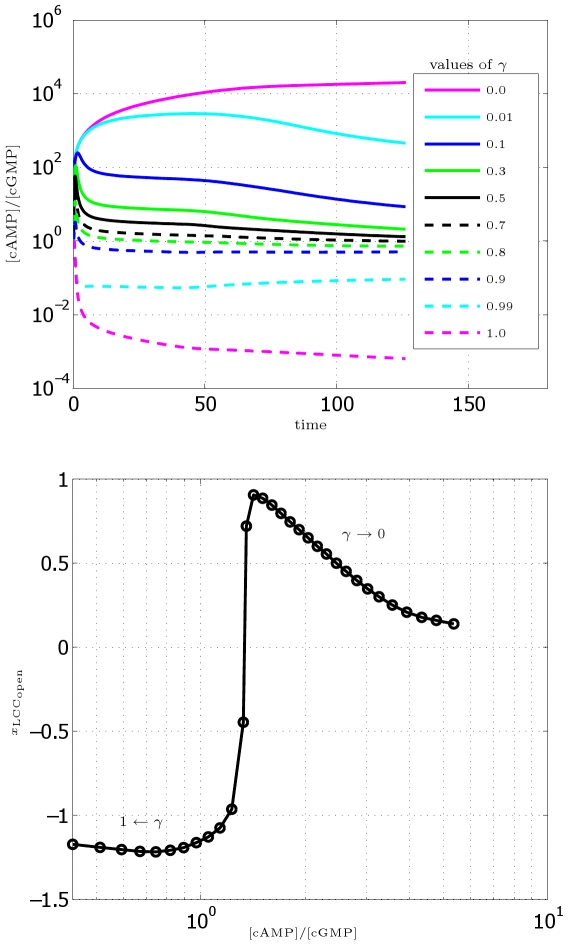
Study of the effect of the [cAMP]/[cGMP] ratio. Top: [cAMP]/[cGMP] ratio as a function of time (in minutes) for different values of 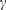 (reported in the legend). Bottom. Position 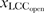 of the abscissa of the barycenter of the open calcium channels (in 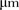) as a function of the ratio [cAMP]/[cGMP] after 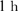. Each marker corresponds to a simulation carried out with a different value of 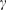.

Using the DCC-UNC5 model, we can analyze more in detail the role of second messengers cyclic AMP and GMP (activated by DCC and DCC–UNC5 pathways, respectively) in modulating the response. We explore a large set of values of 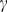 in the range 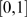 and we compute from each corresponding simulation the 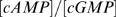 ratio as a function of time (see Fig. 14, (left)). Moreover, in Fig. 14 (right), we show the position of the barycenter of the open calcium channels as a function of the [cAMP]/[cGMP] ratio after 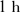. The position of the barycenter indicates here the direction of incipient motion and thus the type of response. These results quantify the idea of Nishlyama-et-al-2003, where the chemotactic response (experimental measure of the axon turning angle) is qualitatively connected to the 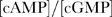 ratio. In particular, the model quantitatively explains the presence of extremal points in the curve reported in [28, Fig.4c] as the outcome of the synergistic interaction of receptors, which enhances the polarization produced in the single receptor case, and of the characteristics of the internal amplification process, which is nonlinear.

## Discussion

We have proposed a mathematical model to study the polarization phenomenon triggered by the exposure of GCs to an external cue, taking as a paradigm the *in vitro* chemotactic assay. The key hypothesis is that symmetry breaking occurs as early as at the level of transmembrane receptors, which undergo a biased distribution after exposure to the cue. This finding appears in the recent work [19], (see also the analysis of [42]), where experimental evidence of such a process is provided and an idea is proposed of an autocatalyzing loop connecting receptors and downstream actin dynamics. Our main contribution is to quantitatively demonstrate via the mathematical model how such a loop is able to achieve GC “front–back” polarization. More precisely, we assess the fact that spatial bias in receptors is a precursor necessary event for chemotactic response, so that upstream gradient sensing and downstream cytoskeleton dynamics cannot be decoupled. Moreover, we analyze further crosslinked effects and, namely, the contribution to polarization of internal enzymatic reactions, which entail activation of a one–to–more production of molecules. The model shows that the enzymatic efficiency of such reactions must overcome a threshold in order to produce a sufficient amplification, which is another fundamental precursor step for obtaining polarization. A simplified version of the model is used to provide preliminary indications about time scales in the front and back processes, via eigenvalue tracking. The findings suggest that the two processes take place with different speeds, the front one being faster than the rear one. This element could play a significant role, even if further investigations should be carried out, A preliminary analysis of the role of diffusion vs. convection is also sketched, establishing the nature of a strongly advection–dominated system. Eventually, we have proposed an extension of the model to address a peculiar behavior arising when two families of receptors interact to produce the response to the same cue. The study of this case allows to propose some ideas on the mechanism of chemorepulsion, as the synergistic interaction of pathways, that contribute to better understand this less studied phenomenon.

The present model demands for improvement. We have run simulations considering a time dependent gradient of ligand. The laboratory experiments (among the very few on this issue for neural cells) reported in [19, Fig.1g], show reversibility of the distribution of the receptors, upon removal of the gradient. Our model tested under these conditions tends instead to maintain the receptor asymmetric configuration and removal of the cue itself is demanded. This fact has led us to conjecture the need for introducing inhibitory effects on receptors. At present, we have indeed assumed that the GC has a pool of receptors capable at each time to bind the ligand and we have not considered their physiological cycle, comprising deactivation by phosphorylation and recycling back in the membrane (as done for example in [29]). Attempts have been made to consider such processes, establishing a life–time for each receptor, after which the receptor is removed and substituted by another one without bias in its angular position on the membrane (keeping in this way the total number of receptors constant in time). Including these effects in the model does not seem to significantly change the results, but deeper investigations on these ideas should be carried out, possibly supported by the availability of further information from the biochemical viewpoint (as done in the context of nonneural cells, for example, in [43]).

## Methods

### Initial and Boundary Conditions

To derive initial conditions for the mathematical model, which are not readily available from experiments, we start assuming the total number of molecules per cell (indicated with the subscript 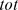) of the following species to be conserved during the integration time, leading to, for all 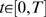


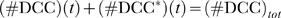
(7a)

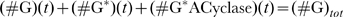
(7b)

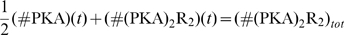
(7c)

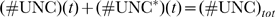
(7d)

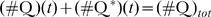
(7e)

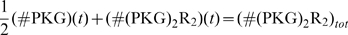
(7f)

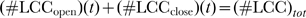
(7g)Total values used in the simulations are reported in Tab. 3.

**Table 3:** Total values of species concentration.

Parameter	Value (#/cell)	Ref.
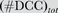	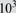	[11]
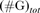	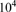	[46]
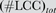	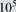	
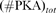	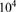	
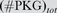	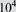	
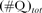	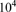	[46]
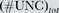	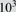	[11]

Moreover, we assume that:

- at time 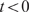, the GC is in a homogeneous state, corresponding to the absence of chemoattractant concentration. This implies that all receptors are in a free state
- adenyl cyclase has an initial value which represents a small fraction of the corresponding conserved total quantity. This is needed to trigger time evolution of the corresponding equation
- at the initial time calcium channels are assumed to be half in open and half in close state.

Using assumptions (1),(2) and (3), we recover from the total values the corresponding concentrations at the left hand sides of Eqs. (7a–g) at 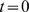. Values are in Tab. 4.

**Table 4:** Initial value of species concentration.

Parameter	Value (#/cell)
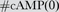	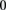
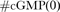	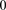
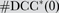	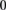
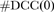	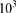
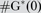	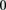
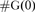	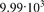
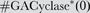	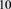
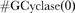	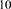
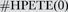	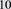
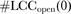	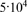
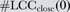	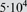
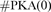	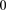
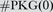	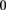
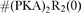	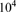
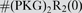	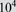
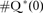	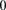
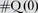	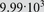
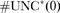	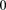
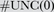	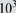

As for the boundary conditions, since the GC is modeled as a circular structure, concentrations and fluxes must coincide at 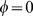 and 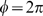. Thus, for each species 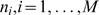, we impose the periodic boundary conditions
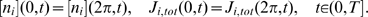



### Modelling Assumptions

#### Unknown chemical processes

At the best of our knowledge, the chemical mechanism which gives HPETE from 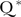 is not known yet. In any case, we can suppose that a multistep reaction is taking place. We denote by X its (unknown) intermediate products. If 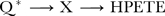, the mass action law gives
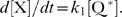
Based on the fact that the processes responsible of the formation of 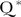 are much faster, we suppose that its production rate can be considered constant, that is 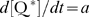, which gives 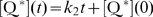. Under such hypothesis and setting 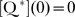 (see the previous section), we have
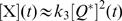
and, eventually
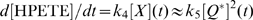
which gives the form in Eq. (5e).

#### DCC-UNC5 receptor complex dynamics

In literature it has been shown (see for example [24]) that UNC5 in order to initiate the repulsive response is to be coupled with a DCC receptor and that both must be ligated to a cue molecule, that is in active form. If one wants to add a detailed model of receptor–receptor interaction, the following reaction should also be considered
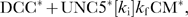
where 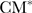 denotes the bound DCC–UNC5 complex. This leads to the following set of kinetic equations for the receptor dynamics
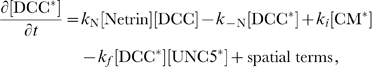
(8a)

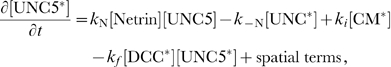
(8b)

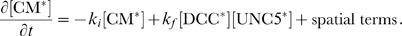
(8c)Then, one should consider 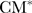 as the species triggering the production of cGMP. The above equations reduce to the model we have proposed in this work by supposing that 1) all the ligated UNC5 receptors bind to 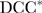 receptors; 2) binding of the complex is instantaneous, which amounts to consider that the formation of the complex is always at the equilibrium; 3) the percentage of DCC receptors coupled with UNC5 is prescribed a–priori using the parameter 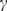, that is we neglect the dynamics given in the equation for 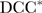 the term 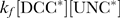. Using the complete model of the receptor dynamics is interesting but too complex to be addressed immediately, above all to give a correct biological interpretation of the results.

### Kinetic Constants

Several kinetic constants entering the model are available from literature references for the same reactions we are dealing with or for very similar reactions. The value of the other parameters has been estimated, based on the following considerations:

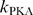
 and 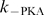: in [44], the half-life time for the exchange
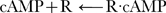
is evaluated to be 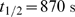. Moreover, in the same paper it is observed that in presence of the catalytic subunit 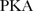, as in our case, the process
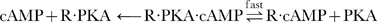
is characterized by a significantly reduced 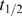. We assume here 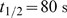, so that
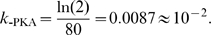
To estimate the direct kinetic constant 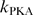, we then use
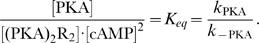
In [29], the concentration of cAMP reaches 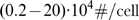 Considering the maximum value, we impose that at the equilibrium the 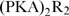 conversion is almost total with a yield of 80–90%. We get
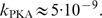
We observe that the order of magnitude of cyclic AMP concentration attained with our model is comparable to the one of [29], used for estimation of this parameter.

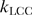
 and 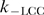: as already mentioned above, in absence of stimulation we have assumed equipartition of open and close channels, which implies
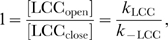
and thus 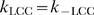. We have chosen 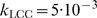, based on a kinetic with speed comparable to the other processes.

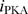
: we use the following relations, which establish the equilibrium of the channels on the front side, on the rear side and the ratio of open channels between front and rear, respectively yielding
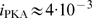

4. DCC–UNC5 pathway: kinetic constants are not easily accessible from literature. Thus, where possible, parameters have been chosen based on analogy with the DCC pathway. Moreover, we have assumed
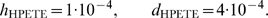

Tab. 5 summarizes the value of the kinetic constants used in the model.

**Table 5:** Kinetic constants.

Parameter	Definition	Value	Dimension	Ref.
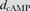	cAMP hydrolysis rate	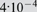	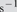	[29]
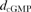	cGMP hydrolysis rate	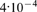	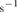	[29]
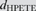	HPETE degradation rate	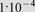	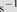	
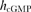	cGMP synthesis rate	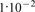	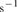	[29]
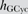	GCyclase activation rate	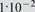	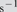	
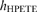	HPETE synthesis rate	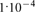	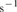	
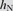	ligand-UNC complex binding rate	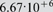	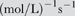	[47]
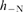	ligand-UNC complex unbinding rate	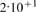	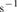	[47]
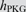	PKG synthesis rate	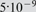	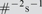	
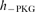	PKG deactivation rate	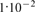	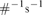	
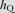	G–protein synthesis rate in UNC pathway	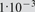	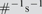	[47],[48]
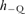	G–protein deactivation rate in UNC pathway	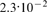	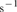	[47],[48]
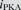	enhancement factor due to PKA	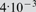	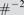	
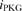	enhancement factor due to PKG	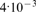	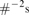	
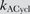	ACyclase activation rate	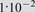	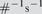	[29]
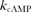	cAMP synthesis rate	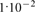	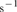	[29]
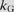	G–protein synthesis rate in DCC pathway	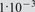	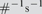	[46]
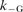	G–protein deactivation rate in DCC pathway	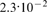	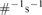	[46]
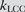	LCC opening rate	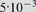	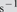	
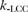	LCC closure rate	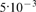	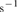	
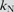	ligand-DCC binding rate	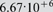	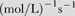	[47]
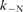	ligand-DCC unbinding rate	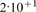	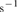	[47]
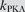	PKA synthesis rate	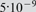	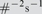	[49]
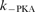	PKA deactivation rate	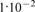	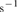	
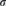	amplification factor	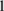	dimensionless	[29]

**Figure 15:**
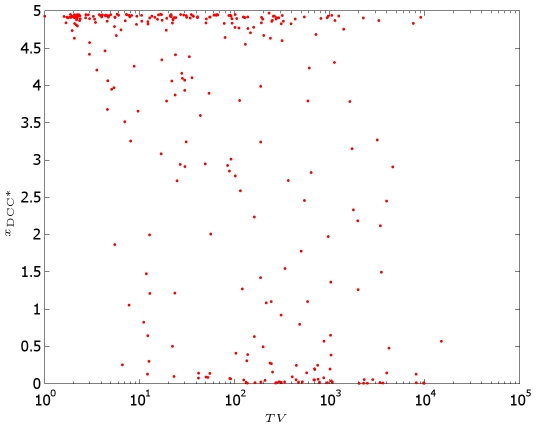
Scatter plot of the position of the barycenter of bound DCC receptors (in 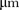) as a function of the total parameter variation. Perturbation in the barycenter displacement is lower than 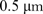 in more than 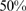 of the tests.

We conclude this discussion exploring the overall influence of the parameters over the model predictions. To do this, we consider the DCC model and we perform a set of 300 trial tests prescribing a random variation of all kinetic constants. We monitor the displacement of the barycenter of the bound DCC receptors after 2 h. The results are shown in Fig. 15, where on the 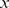 axis we report the total parameter variation 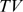 computed as 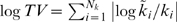, 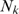 being the total number of parameters, 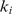 the value of the 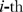 parameter and 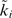 its perturbed value. A 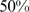 percentage of the tests show a perturbation in the barycenter displacement lower than the 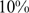.

#### Diffusion and feedback coefficients

The following choices have been made:

1. membrane diffusion coefficient 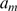: we have taken as a reference the value considered in [7], 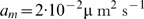. The same coefficient is used for all the membrane species
2. cytosolic diffusion coefficient 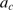: we set 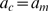. In the context of eukaryotic cells, see [9] for a similar choice, while see [7] for a model with 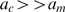. The same coefficient is used for all the cytoplasmatic species
3. feedback coefficient 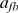: there are no available data. Based on the plausibility of the results, we have chosen 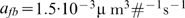.

Tab. 2 summarizes the value of the above coefficients.

### Simulation Algorithm

Both DCC and DCC–UNC5 models constitute nonlinear time dependent diffusion–advection–reaction systems of partial differential equations. Their numerical approximation is a very challenging task: to start with, we partition the GC perimeter into 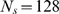 angular sectors (see Fig. 1) and we discretize the spatial derivative operators using finite differences, with a node collocated at the center of each sector. Observe that conservation relations like Eqs. (7) do not hold sector by sector, but they rather apply to the integral on the angle (which represents the number of molecules per cell). Attention must be paid to the fact that the feedback terms in Eqs. (4a) and (5a) dominate the diffusive terms. To avoid spurious oscillations, upwind finite differences or a sufficiently fine discretization should be adopted [45]. Once finite difference discretization is carried out, a system of coupled nonlinear ordinary differential equations is obtained. Due to the different speeds in the reaction dynamics (refer for this issue also to the Results and Discussion), these systems are very stiff and require an implicit time integrator. We have adopted the ode15s Matlab routine with adaptive choice of the time integration step. Numerical evaluation of the Jacobian matrix has been used for the linearization.

The Matlab software package developed by the authors can be made available upon request.
